# The Cost of Caregiving: The Disproportionate and Invisible Impact of COVID-19 on Women

**DOI:** 10.1177/08861099251317493

**Published:** 2025-02-09

**Authors:** Jane Elizabeth Sanders, Emily Carrothers, Olivia Gamble, Derek Neeb, M.K. Arundel

**Affiliations:** 1 The School of Social Work, 113609 King's University College at Western University Canada, London, Ontario, Canada

**Keywords:** Feminist theories and research, gender/Identity, health and well being, mental health, qualitative

## Abstract

The COVID-19 pandemic had a global impact, altering how society and those within it function. While these changes affected almost everyone, there is evidence that women were disproportionately impacted. This reflexive thematic analysis included data gathered from parents, social work students, university faculty, and school board professionals (n = 113) from 2020 to 2023. We explored the experiences of female-identified parents and caregivers during COVID-19. The findings underscore the intense but often invisible burden that women, specifically mothers and caregivers, experienced throughout the pandemic. We found that women experienced acute stress in the realms of paid and unpaid labour, childcare, children's academics, and family mental health. Implications include a call to acknowledge this disproportionate impact and its consequences for gender equity, a recognition of the ongoing impact of COVID-19 on children, youth, families, and women, a recognition of the invisible labour that often falls upon women, and the implementation of critical methodologies in social work education and research to foster the use of such approaches within the field.

## Introduction

During the COVID-19 pandemic, the home became a workplace, school, and virtual meeting space (Smith, 2021). Around the globe, caregivers to school-aged children, more often women (Craig & Mullan, 2011; Eriksson, 2019), expanded their responsibilities to include roles as teachers, early childhood educators, counsellors and more (Del Boca et al., 2020; Sevilla & Smith, 2020). The mental and emotional burden that women, particularly mothers, already experienced was therefore further exacerbated by the additional demands that the pandemic placed upon them (Blundell et al., 2020; Carli, 2020; Dean et al., 2022; Power, 2020). These new roles and the associated unpaid mental, physical, and emotional labour performed by women in the home have traditionally been overlooked as critical resources to maintain our economy and workforce, with unique implications during COVID-19 (Heward-Belle et al., 2022; Sánchez et al., 2020).

The current study, conducted in Ontario, Canada, details findings from a qualitative reflexive thematic analysis (TA) of data from 113 participants across three years of the COVID-19 pandemic. This analysis found that women were disproportionately impacted during COVID-19 and the associated restrictions. However, what was unexpected was that even after detailing the impacts of the pandemic, the women in our study often refuted that their experiences were related to gender. As the effects of the pandemic continue, acknowledging the disproportionate impact it had on women is essential to curtail the growing gender divide.

### The Financial State of Gender Equity

Before COVID-19, women were known to earn approximately 69 percent of the average salary of men (Bonikowska et al., 2019). Globally, the arrival of children, termed the motherhood gap, family gap, or child penalty, drives a long-term wage gap that has increased in the three to four decades before the pandemic and is transmitted across generations to daughters (Kleven et al., 2019; McGinn et al., 2019). In Canada, the location of the current study, women's earnings decrease by 49 percent the year after a child is born; ten years after the birth it is still 34 percent lower than before the birth, while the father's salary remains unaffected (Connolly et al., 2023). Because childcare responsibilities remain largely unequally distributed across heterosexual couples, women more often look for part-time employment and increased flexibility, leading to lower wages, less stability, and a decreased likelihood of bonuses and promotions (Connolly et al., 2023). These penalties persist for years, and they are particularly evident if women have multiple children, have lower education, or are immigrants, and these penalties are often further exacerbated if a couple separates (Le Bourdais et al., 2016; Connolly et al., 2023; Lightman, 2024).

### The Gendered Pandemic

As we learned more about ways that women and mothers bore a greater share of the costs of the pandemic, COVID-19 became referred to as the “gendered pandemic” (Nagy et al., 2023 p. 1072). Cross-nationally, women were already responsible for the lion's share of housework and childcare before the pandemic (Craig & Mullan, 2011; Eriksson, 2019), and this only increased during subsequent lockdowns (Del Boca et al., 2020; Sevilla & Smith, 2020). Households became responsible for what had previously been the purview of the public sphere, including schooling, recreation, and the socialization of children outside of the home. Mental health concerns for youth increased while support decreased, leaving families with little support or recognition (Kourgiantakis et al., 2022). These added responsibilities were in addition to new realities related to employment. For many mothers, this meant juggling work from home with children's daily needs, balancing in-person work with no childcare, and for some, this even meant unemployment and financial peril (Del Boca et al., 2020; Dempere & Grassa, 2023; Sevilla & Smith, 2020). The cumulative effects of this time have been well-documented, including the significant impact on parent and caregiver mental health (Cowie & Myers, 2021; Fontanesi et al., 2020; Javed et al., 2020). Moreover, COVID-19 saw a sharp increase in gender-based violence, femicide, ethnic and race-based violence, and poverty (Heward-Belle et al., 2022; LaBrenz et al., 2023; Quinn et al., 2023; Sánchez et al., 2020). Importantly, those who face multiple forms of marginalization, notably those who are elderly, have ethnic minority status or were experiencing socioeconomic disadvantage, were at even greater likelihood of being negatively impacted by COVID-19 (Ali et al., 2020).

### Discourses of Gender and Caregiving

Dominant discourses, the ideas, beliefs, and narratives that are widely accepted and perpetuated in society, present motherhood as a noble duty that is the only way for women to feel fulfilled. This view is often presented in opposition to the women's liberation movement. This dichotomy is short-sighted, however, as there is room for the profound positives of motherhood while also acknowledging the need for equity and balance (hooks, 1984). Moreover, the ubiquity of this narrative leaves little room for variability within the domestic sphere of life and caregiving tasks are expected to be performed by women, regardless of their employment status (Almeida et al., 2020; Ralli et al., 2021). Policies, social expectations, and workplace culture compound these expectations, creating an environment that is challenging for female-identifying caregivers to navigate. For example, the way the concept of care has been defined devalues caregiving and domestic labour, locating these within the highly gendered context of “women's” work (Nadasen, 2021). bell hooks (1984) argued that female caregiving and domestic labour “should receive deserved recognition, praise, and celebration within a feminist context where there is renewed effort to re-think the nature of motherhood, to make motherhood neither a compulsory experience for women nor an exploitative or oppressive one” (p.136). It is also essential that we honour the intersectional identities of individuals based on their unique positionalities. In unpacking societal views of caregiving and gender, the influence of individual subjectivity is uncovered, illustrating that racism and patriarchy have continued to impact feminist discourses and communities (Beck, 2021; Crenshaw, 1990).

### The Emotional Cost of Caregiving

Before the pandemic, women experienced higher psychological and social distress due to occupying traditionally socio-politically and socioeconomically disadvantaged positions (Almeida et al., 2020; Ralli et al., 2021). COVID-19 was particularly difficult for mothers, during which time there was an increase in clinically significant anxiety and depression, particularly for those with financial stress, those who had difficulties balancing homeschooling and work responsibilities, and those who experienced increased family conflict (Zalewski et al., 2023). Unpaid work time was also much higher during the pandemic lockdown, particularly for mothers, although fathers also experienced an increase in unpaid childcare work (Craig & Churchill, 2021). Women were expected to carry the majority of the caregiving, regardless of their partner's work situation, whereas men's caregiving responsibilities depended on their wife's workload (Sevilla & Smith, 2020). When mothers took on all or most of the childcare, both their well-being and job performance outside of the home suffered (Shockley et al., 2021).

COVID-19 compounded long waitlists and exacerbated existing service gaps for children and families in Ontario. Before the pandemic, four in ten parents reported not receiving adequate or timely help for their children (Tong & McLeod Macey, 2017). COVID-19 caused significant disruption to social services in Canada, including decreases in fundraising, rising operational costs, and fewer in-person services (Deitrick et al., 2020; Prentice et al., 2020). Reduced services led to decreased support when many families were in crisis (Hosseinzadeh et al., 2022; Prime et al., 2020). The burden of care then fell to families, with a quarter reporting missing work to care for their children's mental health (Tong & McLeod Macey, 2017). Alongside societal expectations that mothers provide the bulk of “domestic” labour, many women experienced a decrease in mental well-being, disproportionate decreases in at-work rates, and increased social isolation (Almeida et al., 2020; Robson et al., 2022). With the closure of schools to in-person learning for over 20 weeks in Ontario, these drastic changes to family functioning became particularly difficult (Gallagher-Mackay et al., 2021).

Despite the strength of the literature illustrating the gendered equity issues emerging throughout the pandemic, the current research found that women were not necessarily aware of the ways they were impacted. The unequal burden of the COVID-19 pandemic on women was particularly evident in the new roles they were required to undertake, the stress associated with these roles, and the psychological and emotional impact that these increased expectations had; however, these appeared to be invisible to those most affected.

### Theoretical Foundation of this Research

We have used feminist standpoint theory, a critical theory interested in “relations between the production of knowledge and the practices of power” (Harding, 2004, p. 1) as an analytic framework that explicates a commitment to acknowledging, analyzing and drawing on power and knowledge relationships. This approach is consistent with social work values as both focus on change that facilitates social justice. Feminist standpoint theory encourages a focus on women's lived experiences, particularly experiences of caring work, as the beginning of scientific enquiry (Harding, 2004). Moreover, feminist standpoint theory is used as an epistemic approach to highlight knowledge production that is not only situated with the researchers. Feminist researchers aim to trouble our understanding of knowledge production and as such the very methodological approaches that we use must also be different than the more traditional positivist forms of inquiry. Feminist researchers such as Ahmed (2017), Harding (2016), Sprague, (1998) have not merely argued for the centrality of experiences and emotions in research but have been self-reflexive, self-critical and constantly developed methodologies that create space for the researcher's subjectivity to be accounted for and included in the research process, including the reporting of the findings produced as a result of this form of inquiry. Our alignment with standpoint theory compliments our choice of reflexive TA as our research methodology (Braun & Clarke, 2021).

### The Context of the Current Study

In response to the widespread limitations brought on for public safety during the COVID-19 pandemic, The Support and Aid to Families Electronically (SAFE) program was developed by the School of Social Work at King's University College at Western Canada (King's) to deliver virtual services for parents and caregivers of school-aged children (Sanders et al., 2022, 2023) who are referred through the partnering school boards. King's is a small liberal arts institution with an enrolment of 3,500 students located in London, Ontario, with a population of approximately 511,000. The SAFE program is essentially a mini social work agency with services delivered by students in practicum and supervised by a registered social worker hired by the School of Social Work with no cost, no waitlist, and no limit on sessions (Sanders et al., 2022, 2023). Referrals to SAFE were predominantly mothers, and over half were from the lowest two categories of socioeconomic status based on postal code data. The SAFE program has continued beyond the initial pilot year, and services have expanded to include a second school board. Research was incorporated at the outset.

### The Current Study

The objective of the current qualitative study, using reflexive TA (Braun & Clarke, 2021), was initially to explore the impact of COVID-19 on families who participated in the first three years of the SAFE program. The identified research question was: how were families impacted during COVID-19 and the associated restrictions? By engaging directly with families and those who were supporting them during the pandemic, we obtained unique insights into the impact of COVID-19. An unanticipated finding is the focus of this paper, specifically the invisible impact on women during the first three years of COVID-19.

## Methods

The current analysis is part of a larger multiyear study of the SAFE program examining feasibility of SAFE, educational outcomes for social work students placed there, and the impact of COVID-19 generally. These research aims informed the study design, sampling frame and measures (Sanders et al., 2022, 2023). Reflexive TA of semi-structured qualitative interviews, focus groups, and qualitative Qualtrics surveys was used to allow opportunities for participants to express their experiences with COVID-19 and its restrictions (Braun & Clarke, 2021). TA is a post-positivist reflexive method to organize, analyze, and conceptualize patterns in qualitative data that is well suited to this specific analysis using feminist standpoint theory (Jull et al., 2017).

### Participants

There were 113 participants in the study between January 2021 and August 2023. Year 1 (Y1) ran from January 2021 to August 2021, Year 2 (Y2) from September 2021 to August 2022, and Year 3 (Y3) from September 2022 to August 2023. Four parents in Y1, 13 in Y2, and 20 in Y3 (n = 37) participated in this study. Four professionals from our School of Social Work who were involved in the SAFE program participated in Y1, two in Y2, and five in Y3 (n = 11). Seven social work students placed in the SAFE program participated in Y1, four in Y2, and six in Y3 (n = 17). Finally, 22 school board professionals in Y1, 11 in Y2, and 15 in Y3 (n = 48) participated in the study. Data were collected through individual interviews, focus groups and Qualtrics surveys (see data collection section). Most but not all participants identified as female and White (see Table 1), which closely reflected the population of service users from which the sample was drawn, although representativeness is not a goal of this qualitative study. It is important to note that we use the word “woman” to mean individuals who identify as female and who experienced the world as a woman during the pandemic (American Psychological Association, 2023). We conceptualize family to include “any combination of two or more persons who are bound together over time by ties of mutual consent, birth, and/or adoption or placement” and who take care of each other (The Vanier Institute of the Family, 2024, p. 1).

**Table 1.** Sample Demographics.

Parent Participants	Year 1 (n = 4) Sample (%)	Year 2 (n = 13) Sample (%)	Year 3 (n = 20) Sample (%)
**Gender Identification**			
Female	4 (100%)	13 (100%)	15 (75%)
Male			1 (5%)
Trans woman/feminine/female			1 (5%)
Not Available			3 (15%)
**Race**			
White	4 (100%)	6 (46.15%)	14 (70%)
Middle eastern			3 (15%)
Latin American			1 (5%)
Mixed Indigenous and White			1 (5%)
Not Available		7 (53.85%)	

The SAFE Program Students	Year 1 (n = 7) Sample (%)	Year 2 (n = 4) Sample (%)	Year 3 (n = 6) Sample (%)
**Gender**			
Female	6 (85.71%)	2 (50%)	6 (100%)
Male	1 (14.29%)	2 (50%)	
**Race**			
Black		1 (25%)	
White	5 (71.43%)		4 (6.67%)
White and Indigenous		1 (25%)	
Hispanic			2 (33.33%)
Mixed Black, Greek, French, Scottish		1 (25%)	
Prefer not to answer	1 (14.29%)		
Not identified	1 (28.57%)	1(25%)	

Referring Professionals	Year 1 (n = 22) Sample (%)	Year 2 (n = 11) Sample (%)	Year 3 (n = 15) Sample (%)
**Gender**			
Female	17 (77.27%)	6 (54.55%)	12 (80.00%)
Male	2 (9.09%)		1 (6.66%)
GenderQueer		1 (9.09%)	
Prefer not to answer	1 (4.55%)	1 (9.09%)	2 (13.33%)
Not identified	2 (9.09%)	3 (27.27%)	
**Profession**			
School support counsellor	9 (40.91%)	5 (45.45%)	3 (20.00%)
Social worker	4 (18.18%)	1 (9.09%)	7 (46.66%)
Social worker/attendance counsellor	7 (31.82%)	3 (27.27%)	4 (26.66%)
Not identified	2 (9.09%)	2 (18.18%)	1 (6.66%)
**Race**			
White- Europe or North America	18 (81.82%)	4 (36.36%)	13 (86.66%)
Prefer not to answer	1 (4.55%)	1 (9.09%)	1 (6.66%)
Not identified	3 (13.64%)	6 (54.55%)	
Mixed background			1 (6.66%)

School of Social Work Professionals	Year 1 (n = 4) Sample (%)	Year 2 (n = 2) Sample (%)	Year 3 (n = 5) Sample (%)
**Gender Identification**			
Female	4 (100%)	2 (100%)	5 (100%)
Title/Position			
Administrative assistant	1 (25%)		
Coordinator of field education	1 (25%)		1 (20%)
MSW Practicum Coordinator		1 (50%)	1 (20%)
Field supervisor	1 (25%)	1 (50%)	1 (20%)
Faculty consultant	1 (25%)		2 (40%)
**Race**			
White	4 (100%)	2 (100%)	5 (100%)

### Participant Recruitment

All parents who were referred to the SAFE program from January 2021 to July 2023 were invited to participate. Information on informed consent was distributed to all parent service users of the SAFE program (P) upon termination from the program. Informed consent forms were emailed to all students placed in the SAFE program (ST), all professionals from our School of Social Work involved in the SAFE program (K), and all school board professionals eligible to refer to the SAFE program (SB). Parent participants each received a $25 gift card. Research ethics approval was granted through the supporting university.

#### Researcher Description

The lead author (PI), a cis-gendered, White, middle-aged, highly educated woman, had prolonged engagement through over 25 years of child, youth and family therapy and direct practice and research in school boards. The remaining research team consisted of the SAFE program practicum supervisor, two research assistants who were also MSW students placed in the SAFE program and the Manager of Professional Practicum Education. Therefore, the team consisted of researchers who are knowledge users and service providers. All authors identify as White, three identify as female, one as gender non-binary, and one as male, and three were parenting and/or grandparenting during this period of COVID-19. Reflexivity, applied through a lens of feminist standpoint theory, was incorporated into the study in multiple ways. During data collection, many, if not most, of the participants initially rejected ideas that the impacts of COVID they had just detailed were related to aspects of their social location, notably gender. When probed by the interviewer, informed by their insider female and feminist stance, participants reflected more deeply on this possibility. Data analysis was further informed by the authors’ critical and reflexive application of the methodology with intentional inspection of power, privilege and the ways that social discourse serves to render invisible, even to those affected, the impacts of systemic inequity.

### Data Collection Procedures

Data were collected through individual, semi-structured, in-depth interviews and focus groups each lasting 60–90 min, as well as Qualtrics surveys with open-ended text questions. Interviews were conducted over Zoom and audio recorded with permission. The Qualtrics surveys were distributed to those participants who were interested in sharing their knowledge but were unable to commit to an interview or focus group (P: n = 9 and SB: n = 40). The PI conducted all interviews in the first year except those with SAFE students; in the second and third years, interviews were conducted by a combination of PI and RA. These were professionally transcribed and analyzed along with the qualitative content from the surveys. Interviews began with questions about the impact of COVID-19 and the associated responses and restrictions on parents and families. We began with general questions, including: how did COVID-19 affect you, affect your family, what was the impact on your child's school/educational experience, and how was work impacted (including in-home unpaid labour)? We followed these questions with: “Do you think that any of these impacts are related to you or your family members’ gender, race, ethnicity, sexual orientation, ability, faith/religion/spirituality or anything else?” Questions were adjusted for each of the participant groups. For example, SAFE students were asked, “How do you think COVID-19 impacted families?” rather than “How did COVID-19 affect you…”. Furthermore, the semi-structured and reflexive method and stance allowed for responsive adjustments during each interview.

### Data Analysis

As per the first step of TA, four research team members familiarized themselves with the data. The four subsets of data (P, ST, K and SB) were each line-by-line coded by two independent coders. Parent data were prioritized for line-by-line coding, and data from other participant groups were incorporated in a process consistent with constructivist grounded theory's constant comparison in which there is constant comparison of “data with data, data with code, code with code, code with category, category with category, and category with concept and finally major categories are compared with relevant literature” (Charmaz, 2014, p. 342). The focus was on inductive coding categories. In step three, codes and potential themes were identified for each subset of data, which were then reviewed, and inconsistencies were discussed. In the fifth step, the team collaboratively defined and refined the identified parent themes and co-occurring subthemes of the entire data set. As per step six, the analysis was finalized by the lead researcher whose subjectivity is considered an asset used to structure the analysis in the current study (Braun & Clarke, 2021). The multiple participant subgroups were included as a form of triangulation. To further enhance reliability, trustworthiness, and credibility, an audit trail was kept to document research decisions (Anastas, 2004; Nowell et al., 2017). Each interview, focus group, or survey was coded independently by two research assistants, although the subjectivity of the lead author was viewed as an asset in guiding the final analysis (Braun & Clarke, 2021). This approach, our data collection and analysis, is consistent with Harding's (2016) notion of strong reflexivity which “would require the objects of inquiry to be conceptualized as gazing back in all their cultural particularity and that the researcher, through theory and methods, stand behind them, gazing back at [their] own socially situated research project” (p. 163).

## Results and Discussion

The current analysis exposed ways that COVID-19 increased family stress in several areas, including increased labour demands related to schoolwork, family mental health, and, in many cases, demands from work outside of the home and increased financial stress. While the important stories shared in the current study were not surprising, it was surprising how few of the parent participants initially articulated their experiences as being related to gender. However, the interviews taken over three years were consistent, and throughout the process of our reflexive TA and the application of feminist standpoint theory, it became clear there was a distinct and disproportionate impact based on gender.

It should be noted that while we did not actively recruit female participants, those who agreed to be part of this research were disproportionately female and therefore, our findings are specific to the mostly women who were part of our research. Moreover, it was predominantly the mothers in a household that were referred to the SAFE program's services, ranging from 80% in Y2 to over 90% in each of Y1 and Y3. We view this as an extremely strong indicator of societal expectation that women should be taking care of the mental health of the family. This was corroborated in the data related to referrals, as one SAFE program student advised, “there was only information or contact information listed about a mum” (Y1-I-ST01). From referral to service, it was female parents and caregivers who arranged the counselling session timing, who received emails from the program and the school, and who attended case consultation meetings with the program. As another SAFE program student noted, “most of the people I spoke to were the mothers, and there was an additional struggle with the added role of the caretaker” (Y1-FG1-ST-R03). These extra burdens in the caretaker role during COVID-19 created a significant mental load on the mothers that the SAFE program was supporting. These extra burdens are detailed in the next section.

### Increased Labour During COVID-19

There were several ways that the participants were impacted by the pandemic. Firstly, it was noted that women were more often responsible for the added “invisible labour… emotional labour, mental labour, as well as physical labour” (Y1-FG1-ST-R03). The extra demands were particularly felt when families were isolated from their support systems, as one participant articulated,

We had an immense village of people helping us, to nothing…So, that meant that I had to become the therapist, the OT, the speech therapist, the teacher, the caregiver, the mom. Everything was on my shoulders. It was extremely, extremely stressful. (Y3-I-P44)

One parent participant spoke of the balance that was required to add in the extra labour during the pandemic, “I would be working slightly shorter hours in the morning/afternoon and coming home and then also having to work after the kids went to bed” (Y1-I-P06). Participants also noted the challenge of this outside of the home, “they also had additional unpaid labour at work because of the nature of feminized labour in spaces like office spaces” (Y2-I-K-05).

It was evident in the data that this extra load disproportionately fell to mothers, with one SAFE program student participant noting that “female clients were taking on most of the additional responsibilities that the pandemic placed on families” (Y1-FG1-ST-R03). School board participants felt the impact of this trend, stating, “women are holding everything together in all spaces. And we are exhausted” (Y3-S-SB24). Parent participants stated, “it was such a stressful situation for everyone that I couldn’t even necessarily help them with everything I thought they needed help with” (Y2-I-P12). At least one female parent identified not only the additional burden of childcare but noted that her husband was struggling significantly, which added to her burden as well,

So, he's better, but also, he's one of those people where during times of stress the bad qualities come out very quickly. It was very stressful to try and navigate supplicating him while also making sure I was doing the right thing for the kids. (Y2-I-P12)

Finally, for those in female dominated professions, such as entertainment and hospitality services, a shutdown of their workplace meant that everyone was out of work. This was amplified for women experiencing multiple forms of marginalization. Likewise, many participants were in healthcare, another female dominant sector, and the extra responsibilities of caring for those who were physically vulnerable meant extreme vigilance and isolation practices, as one participant described, “because I was in healthcare, I just stayed home, so that caused a lot of stress, like, a lot of worry…I didn’t want to bring anything to work that could potentially harm a child” (Y3-I-P35).

It is important to note that participants told stories of their husbands being impacted negatively as well, “…he was like, I'm not seeing my family, I'm forced to work more than 40-h a week. He has Crohn's disease, so that affected his physical health as well” (Y3-I-P52). Moreover, a few participants denied experiencing negative effects during COVID-19, particularly if their husbands were either not working or already working from home, “home-wise, I would say I didn’t have to worry labour-wise. We have my mom living with us and my husband was off from work so they were both home able to take care of the house, and then I was just able to work full-time” (Y3-I-P35). However, these experiences were not common in this sample.

Two specific areas of increased labour stood out notably in our analysis. These were being responsible for the academic load during COVID-19 and the mental health of the family.

#### Academic Load for Children Overwhelmingly Fell to Mothers

Parent participants noted that they took on the academic load in their household during lockdowns, highlighting the struggle to maintain academic focus for the children while also working, completing their own schooling, or managing other children in the house. Participants shared, “by the end of the first year with COVID we had pretty much stopped doing most of the stuff that the teacher provided because she just had no interest in it” (Y2-I-P15). A SAFE program student observed, “now there is the triple workday if you’re probably doing more labour around the home, labour outside the home and teaching your child” (Y1-FG2-St-R04). Professionals from our School of Social Work also observed this trend within the home, sharing “moms…already had a whole bunch of unpaid labour, and now they were teachers, or they were daycare providers” (Y2-I-K05).

#### Responsibility for the Mental Health of the Family was Overwhelming

The heavy yet invisible burden of family mental health took a toll on the women in this study. Female-identifying caregivers felt responsible for the well-being of the family and that they must put their needs on the shelf in order to put the rest of the family first. However, this prioritization became unsustainable for many as the pandemic wore on: “this took a toll on me at some points as it became very overwhelming and mentally exhausting to be positive and headstrong and reassuring to everyone else” (Y2-S-P03).

Many women felt that it was complicated to admit they were struggling because they love their children and didn’t consider it a burden to care for them. However, the added responsibilities during the pandemic, coupled with their feelings of unease, fear, and the impacts on their careers was overwhelming, as one parent observed, “I found that my mental health had the greatest impact on the mental health and emotional regulation of the entire household” (Y2-S-P03). One participant reflected on the influence of her self-sacrificing behaviour on her children,

Sacrificing your own self all the time isn’t helpful to your kids all the time…if they just see you being a martyr all the time, then it teaches them to just be martyrs, and I don’t want my children to be martyrs. (Y2-I-P12)

### The Invisible Impact on Women

As described above, participants were asked general questions about the impact of COVID-19. After telling story after story of becoming teacher and mental health support, stories of lost jobs or closed businesses, we asked if they would attribute any of the impacts they had shared to who they were as a person, “so your gender, your sexuality, religion, race, ethnicity, anything?”. Overwhelmingly, parent participants responded, “no”.

Some of the mothers stated they took on more of the burden just because they happened to be the ones who were home during the day. Others stated they took on more of the burden because they just happened to be the ones who were working outside of the home during the day as one articulated,

…whoever was at home…dealing with three children, you’re not really doing any of the household stuff. And that just happened to be my husband who was always home…I’d come home, I’ve worked, I now deal with kids and dinner, and then I’m also doing all of the household things. So, this was burdensome, but again I don’t really think it was because I was the female. I think it was just he was home and that was the way it was. (Y1-I-P06)

Others felt the burden fell to them because they happened to be the ones who were better with certain tasks, although they didn’t see this as a disproportionate impact related to gender generally. Often, the academic load was framed as expertise that the mother held in the family, be it the connection to the school, the mastery of the children's technology, or that they had the time in their day to assist with the children's academics when their partner might not. One participant noted:

I’m more academically inclined and he's not. So, the days he was off, and we were doing schooling, he could get them on, but he can’t really help them with schooling…It was more just the way it worked out best for us. (Y2-I-P23)

Even after detailing the impacts of COVID-19, some parent participants were reluctant to name these as gendered. As an example, one female participant had closed her part-time business as it was overwhelming to maintain with everything else. This participant stated, “my focus then was more on family than working. So, I guess kind of giving up my business, it did help keep me grounded to be more available for my family” (Y3-I-P40). As noted earlier, not only were women more likely to take on family responsibilities and to be responsible for the extra labour brought on during COVID-19, but women are also more likely to look for part-time work and jobs with flexibility, such as the business this participant was running. When asked directly about how gender may have influenced her situation in closing the business, she responded,

Yeah, I did feel like that, because a lot of the trades that were not allowed to work are mostly women oriented, but I did try to look past that. I believe if you want to be treated equally, then you’ve got to fight equally. I don’t think I closed down the business because of being a woman. It was just a lot to handle, and the rescheduling of clients…was just draining on a day-to-day. (Y3-I-P40)

Another participant articulated, “I think in some ways yes, but I don’t know that it's gender, I think it's me” (Y2-I-P23).

Many participants acknowledged the role of gender in how COVID-19 impacted them when probed further by the interviewer, for example, “possibly, because I was doing housekeeping in a hotel so whenever we went into lockdown it got shut right down because nobody would go anywhere. No business, nobody can work, right. For the most part we were all females” (Y1-I-P05). One parent participant tentatively but powerfully explained the gendered impact as follows:

gender has played a little bit of a part…I feel. I could be totally wrong on this, but I think as females we’re already in a society where we are more vulnerable. And I think during COVID and post-COVID-19 that our vulnerability increased the lack of safety. (Y2-I-P16)

The unanticipated finding of not only the disproportionate impact of COVID-19 on mothers but, and perhaps more importantly, the initial invisibility of this impact is a significant finding. This finding further illustrates the ways that the values, norms and dominant structures of society are reproduced without question, rendering their disproportionate impact invisible, even to those who are most affected, making the reproduction of the capitalist mode of production possible (Camps Calvet et al., 2024).

### Impact Over Time

The long-term impact of COVID-19 was perhaps most clearly articulated in the year three data. Throughout the pandemic the stress accumulated: “It was difficult. By June I was so burnt out. I hit probably a very low, low point…And I had never been to that point in my life ever. And it's the cost of all of it. So, it was difficult” (Y3-I-P44). As one participant noted, they were ignoring their own feelings and stresses to focus on the needs of the children, which finally overwhelmed them: “personally I ignore any feeling or stresses for me, and keep focusing with the kids…after the pandemic start[ed] to ease, I [would] feel very stressed. Because all this time you keep pushing, just to keep the balance in this family” (Y3-I-P36). This impact was multiplied for single mothers: “I felt extremely overwhelmed being the only one taking care of my three children” (Y3-S-P01).

Not only did stress build, but opportunities to find work or return to school were suspended during this time, setting women back years, as one woman trying to gain skills after childbirth articulated,

after I did my baby, and she was one or one year and half, I start think about studying to find a new job or do a new thing. But unfortunately, it was hard for me because my little daughter, I can’t find any daycare for her, and I have to stay at home with my children, I can’t leave them. (Y3-I-P36)

COVID-19 did not only impact women; it was substantial for everyone. However, there was a differential pandemic-related impact on women. Women were often subjected to a triple workday consisting of their regular employment, engaging in domestic labour, with the addition of caregiving, child-rearing and education (Jasrotia & Meena, 2021; Peck, 2021; Sharma & Vaish, 2020). Consistent with extant literature, we found an unfortunate association between increased household demands for women and increased physical, emotional, and psychological stress (Jasrotia & Meena, 2021; Sharma & Vaish, 2020). And yet, this impact was largely invisible.

## Key Implications and Contributions

It is important to recognize the ways that events such as COVID-19 impact certain individuals disproportionately and to be aware that this impact can go unacknowledged across society as well as by those who are most impacted. There are, therefore, several implications for this work. Firstly, acknowledging this disproportionate impact is essential to addressing the widening financial gender gap identified in extant literature (Kleven et al., 2019; McGinn et al., 2019). Women were already in a “resource loss” position, and COVID-19 has exacerbated this, jeopardizing progress toward gender equity (Peck, 2021). It is important to note that “women” is not a uniform category; “none of us is unidimensional”, and every female-identified individual will have experienced the impact of COVID-19 uniquely (Goodkind et al., 2021, p. 482).

Secondly, the gender or motherhood gap (Kleven et al., 2019; McGinn et al., 2019) is exacerbated by the additional invisible labour that most often falls to women related to family mental health, as illustrated in the current study. This labour increased during the most critical phases of COVID-19 and mental health services have not returned to pre-COVID-19 availability, which was already shamefully inadequate (Children's Mental Health Ontario, 2020; Georgiades et al., 2019). Lost time in the workplace has a compounding impact on the already significant gender gap that is exacerbated by the increase in children's mental health concerns, which are the primary concern of mothers. Moreover, there is a cost to the mental health of parents who are caring for children along with their own mental health concerns (Acri & Hoagwood, 2015). Parents are generally unlikely to receive services for emotional health, and yet without support, parental mental health concerns can negatively impact families (Acri & Hoagwood, 2015). Therefore, school closures, which have disproportionately impacted low-income families, racialized and Indigenous communities, newcomers, and those with disabilities, are deepening educational inequities as well as physical, mental health, and safety harms for children, with long-term impacts (Gallagher-Mackay et al., 2021). We believe the mental health and educational impact of COVID-19 on children, youth and families will be long standing, which means that the impact on parents, particularly mothers, will be ongoing.

Thirdly, there is every indication that such crises will continue. Therefore, it is essential to acknowledge their disproportionate impact, whether brought on by a family emergency such as hospitalization, a regional environmental disaster such as flooding or fires related to climate change, or a global pandemic. Without a situated perspective that acknowledges the ways that women are uniquely impacted, it is impossible to adequately address gendered expectations for women. This acknowledgement is necessary for societal change that will enable appropriate support for everyone throughout such crises. In particular, restrictions to in-person learning were a significant factor in the disproportionate impact of the pandemic, not only on women but also on children and youth. This research can support the goals identified for making the educational system more resilient to future crises (see OECD, 2021).

Fourthly, it is important to stress that caregiving is often (but not necessarily) important to who women are, and we want to highlight that this should be celebrated and valued while also recognized as energy-intensive. We must focus on ways for families and society to acknowledge the hidden cost of caring, as this is essential to ensure that the gap in financial well-being, mental health, and social status is eradicated rather than exacerbated. Caregiving and domestic labour should be recognized, praised, and celebrated, no longer interpreted as compulsory for women nor exploitative or oppressive. As noted above, there is room for the profound positives of motherhood while also acknowledging the need for equity and balance (hooks, 1984).

Fifthly, this study illustrates the importance of implementing critical methodologies, including critical feminist methodologies in which the subjectivity of the researcher is celebrated and critical interpretive theories are incorporated. As noted by Goodkind and colleagues (2021), “feminist inquiry leads “to the elucidation of visible and obscured dynamics of oppression” (p. 484). Critical theory involves analysis and critique and is considered essential or critical to understanding and addressing inequality and working toward social work's value of social justice (Collins, 2019; Goodkind et al., 2021). In a similar vein as reflexivity, Crotty (1998) writes that “critical inquiry illuminates the relationship between power and culture and, in this picture of things, culture comes to be looked upon with a good measure of suspicion” (p. 158). This aims to widen the lens from individual researcher reflexivity to the situatedness of the project, the participants, and the researcher. Without a reflexive insider and critical feminist stance, the gendered impact of COVID-19 in this study could have been missed within the unquestioned reproduction of dominant discourse (Camps Calvet et al., 2024). We want to reiterate not only the significance of the disproportionate impact of COVID-19 on mothers identified in this study but the unanticipated finding of the invisibility of this impact.

Finally, social work educators must continue to foster critical thinking in all its definitions. This includes the type of critical thinking defined and applied in this paper, with critical attention to the social context, historical, social and political in which we are all immersed (Goodkind et al., 2021). Further, we must encourage the application of theory and practice beyond the individual to fully apply the person-in-environment framework that is celebrated in social work education and practice (Goodkind et al., 2021). This study, emerging from a social work program providing social work services while educating future social workers, strives to meet social work's social justice goals by recognizing the pervasiveness and centrality of power, challenging the unquestioned reproduction of dominant discourses, and the importance of critical subjectivity and collective action for liberation (Camps Calvet et al., 2024; Goodkind et al., 2021; Harding, 2016).

### Strengths and Limitations

Qualitative research does not strive to be representative, and due to the nature of the data collected, only those referred to the program and willing to participate are represented in our study. COVID-19 disproportionately impacted marginalized populations (Salerno et al., 2020; Tai et al., 2022; Webb Hooper et al., 2020), however, the experiences represented in this study are limited given the demographics of those involved in this study. As such, further intersectional research is needed to articulate how multiply marginalized women, for example, sole parent households, Black, Indigenous or racialized minorities, those with disabilities, and immigrant/newcomer parents, were impacted during COVID-19 (Crenshaw, 1990). Additionally, as researchers, we are always working to consider our implicit biases and how our social locations may influence power dynamics, study participants, or study design while acknowledging the relationship that social work and academia have to racism and colonialism (Murray-Lichtman & Elkassem, 2021).

## Conclusion

The pandemic furthered an existing gap and exacerbated the caregiving load that women carry within families, often to the detriment of their mental health (Almeida et al., 2020; Del Boca et al., 2020; Robson et al., 2022; Sevilla & Smith, 2020). Yet, due to the hegemonic ideas that we are all socialized to believe, even those directly impacted might not recognize this as a gendered issue, fostering self-sacrifice and silence in this burden (Budgeon, 2014). It is critical that we acknowledge these gendered concerns to address the resulting harms to people of all genders, to address the widening gender gap, to prepare for an impending increase in local and global crises, to foster policies and programs that support caregivers, to engage critical theory in research and education to dismantle binary systems, and to create lasting, meaningful change.
